# Exploring toroidal anvil profiles for larger sample volumes above 4 Mbar

**DOI:** 10.1038/s41598-024-61861-2

**Published:** 2024-05-18

**Authors:** Claire C. Zurkowski, Jing Yang, Francesca Miozzi, Suzy Vitale, Earl. F. O.’Bannon, Zsolt Jenei, Stella Chariton, Vitali Prakapenka, Yingwei Fei

**Affiliations:** 1 Earth and Planets Laboratory, Carnegie Institution for Science, 5241 Broad Branch Road, NW, Washington, DC 20015 USA; 2 Present Address: Lawrence Livermore National Laboratory, 7000 East Ave, Livermore, CA 94600 USA; 3 Center for Advanced Radiation Sources, The University of Chicago, 9700 South Cass Avenue, Building 434A, Argonne, IL 60439 USA

**Keywords:** Planetary science, Solid Earth sciences, Materials science, Physics

## Abstract

With the advent of toroidal and double-stage diamond anvil cells (DACs), pressures between 4 and 10 Mbar can be achieved under static compression, however, the ability to explore diverse sample assemblies is limited on these micron-scale anvils. Adapting the toroidal DAC to support larger sample volumes offers expanded capabilities in physics, chemistry, and planetary science: including, characterizing materials in soft pressure media to multi-megabar pressures, synthesizing novel phases, and probing planetary assemblages at the interior pressures and temperatures of super-Earths and sub-Neptunes. Here we have continued the exploration of larger toroidal DAC profiles by iteratively testing various torus and shoulder depths with central culet diameters in the 30–50 µm range. We present a 30 µm culet profile that reached a maximum pressure of 414(1) GPa based on a Pt scale. The 300 K equations of state fit to our *P–V* data collected on gold and rhenium are compatible with extrapolated hydrostatic equations of state within 1% up to 4 Mbar. This work validates the performance of these large-culet toroidal anvils to > 4 Mbar and provides a promising foundation to develop toroidal DACs for diverse sample loading and laser heating.

## Introduction

Generating ultra-high pressures under static compression is critical to probing planetary interiors and exploring exotic chemistry and physics phenomena. At present, the NASA Exoplanet Archive confirms over 5000 identified exoplanets (https://exoplanetarchive.ipac.caltech.edu/). Of the known exoplanets, a significant portion of them exist in the Earth-to-Neptune size range and have widely varying densities^1–3^. Probing the interiors of Earth-to-Neptune-sized planets requires capabilities to reach pressures between 3 and 6 Mbar^4,5^—conditions beyond the capabilities of conventional static compression techniques in the DAC. Characterizing the materials that form in the interior of these bodies also requires capabilities to probe reactions, phase transitions, and density changes in complex ice-silicate-metal systems at high temperatures and pressures. Reaching these ultrahigh pressures is also critical for probing exotic physical and chemical properties, such as metallization^6,7^, superionicity^8^, superconductivity^9^, electride formation^10,11^, and structural deviation from closed-packing^12^.

In the past decade, novel modifications of the standard diamond anvil into the toroidal and double-stage configurations have advanced static compression capabilities into the 0.4–1 TPa pressure range^13–22^. The toroidal diamond-anvil design consists of a toroid shape around a central culet typically milled with a gallium or plasma focused ion beam (FIB). This shape effectively traps gasket material to support the culets, accommodates anvil deformation, and lowers the shear stresses in the diamonds under ultrahigh pressure conditions^19,20^. Toroidal anvils with 8–16 µm culet diameters have reached pressures of 6 Mbar^19,20^, and successive studies using these anvil shapes with culet diameters up to 25 µm can achieve pressures between 4 and 5 Mbar^7,22,23^. The double stage design consists of a standard anvil with an added secondary anvil made of nanocrystalline (NCD) or nanopolycrystalline (NPD) diamond synthesized in the multi-anvil press^13–18^. This design offers confining pressure from the sample chamber of the first-stage anvils. This confining pressure both supports and changes the yield strength of the secondary anvil enabling it to support higher loads. Pressures between 5 and 10 Mbar have been reported using anvils with secondary stages ranging from 3 µm culet diamonds to hemispherical anvils^13–18^. Additionally, pressures up to 1 TPa have been reported by applying a double stage NCD hemisphere onto a toroidal anvil^14^. A recent study further reports that secondary stage anvils with a toroidal shape placed on standard anvils have the most stable double stage configuration of the current shapes tested^21^.

These previous toroidal and double-stage anvil studies have primarily focused on reaching the maximum possible pressure in the DAC and attempts to heat the sample or load the sample embedded in thermal insulation are limited^13,14,19^. Probing the interiors of super-Earths and sub-Neptunes not only requires the generation of 3–6 Mbar pressures, but it also requires synthesis of materials at high temperatures in complex petrology regimes. Here we present an expanded study of toroidal anvil profiles that accommodate large enough sample volumes for multi-layer sample packages and synchrotron or laboratory laser heating specifications. The toroidal anvil designs tested here have 30–50 µm culet diameters. After modifying the culet shape, torus depth, and shoulder depth to optimize the attainable pressures with these anvils, we present a profile that reached a maximum pressure of 414(1) GPa based on the Pt scale^24^. We validate the performance of these anvils by measuring the volumes of gold and rhenium, fitting an equation of state, and comparing the fits to previous studies. Going forward, further development of the anvils, sample configuration, and heating methods may be tested for routine high temperature experiments, as the 12–18 µm diameter sample chambers tested with these anvils offer new possibilities for experiments with diverse samples at extreme pressures and temperatures.

## Results

### Toroidal anvil design optimization

**Figure 1:**
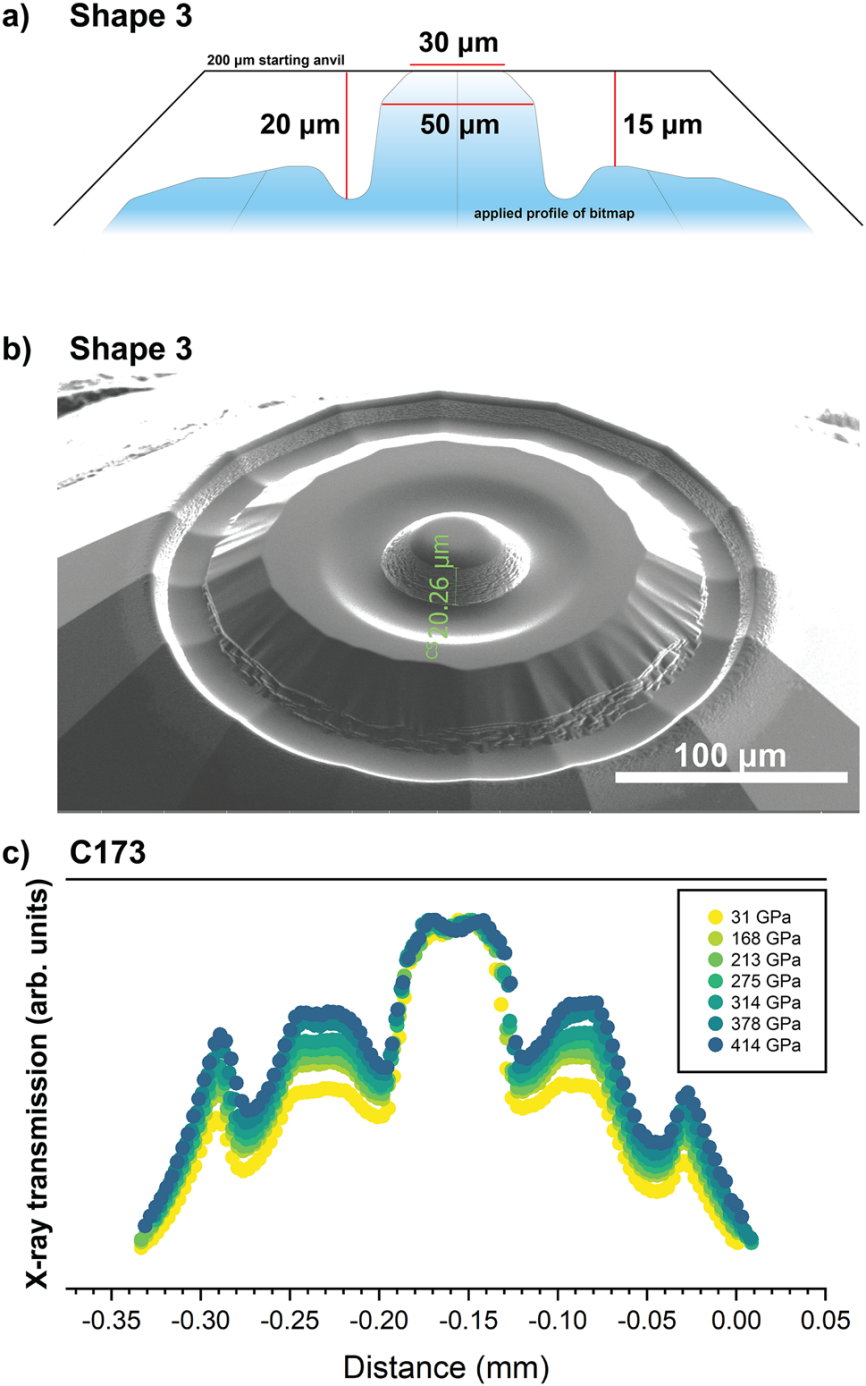
(**a**) General schematic of the toroidal Shape 3 tested in this study on sample C173. The profile of the starting 200 µm standard anvil is included. The idealized bitmap profile, shaded in blue was scaled to 250 × 250 µm, such that the outer shoulder overlaps with the pavilion of the standard anvil. A sharp edge is produced at the boundary of the bitmap and is an artifact of the differing slopes between the bitmap and the diamond pavilion. (**b**) Example of the toroidal anvil Shape 3 milled with the focused ion beam showing the real profile that results from milling onto the pavilion and the sharp outer lip produced by the bitmap slope that is offset onto the pavilion. (**c**) Absorption scan of sample C173 with Shape 3 between 30 and 414 GPa measured at the center of the sample chamber based on the Pt scale^24^.

The starting anvils for each test were standard cut with 200 µm diameter culets. Toroidal shapes were milled with the focused ion beam incrementally modifying the culet diameter, torus depth, shoulder depth, and bevel of the central culet. As the behavior of toroidal anvils with varying specifications for culets larger than 25 µm has not been well characterized, we initially scaled the culet and torus-depth proportions linearly from (19) and (20) to a 30–50 µm culet. With this, we tested toroidal shapes with torus depths in the range of 9–20 µm (Fig. 1) and with shoulder depths ranging from 2/3 to 3/4 the torus depth^20^. Specific details for each anvil tested are provided in Table 1. The various proportions tested are divided into three shapes: Shape 1 contains a 50 µm culet with a shoulder depth that is 2/3 the torus depth, Shape 2 consists of a 50 µm culet with a shoulder depth that is 3/4 the torus depth*,* and Shape 3 consists of a 30 µm culet that bevels to 50 µm and a shoulder depth that is 3/4 the torus depth. A schematic of these three tested anvil shapes is provided in Figure S1 and the anvil shape that performed to the highest pressures in this study is shown in Figure 1a.

**Table 1:** Toroidal anvil specifications and performance evaluated for each sample.

Shape	Sample	Culet (c)/bevel (b) (μm)	Torus depth (t) (μm)	Shoulder depth (s) (μm)	Loading	Maximum pressure (GPa)
1	C149	50	9	6	Au	165
	C160	50	12	8	Pt + Au	169
2	C161	50	15	11.25	Pt + Au	185
	C164	50	18	13.5	Pt + Au	211
3	F001	30/50	16	12	Pt + Au + Fe–C	200
	C172	30/50	18	13.5	Pt + Au + SiO2	249.2(3)
	C173	30/50	20	15	Pt + Au	414(1)
	C30	30/50	20	15	Pt + MgO	310
	C117	30/50	20	15	Pt + Au + Fe–C	270
	F002	30/50	20	15	Pt + W	350

The torus depth and shoulder depth are approximated from the measurement of the torus depth – culet-bevel in cross-section in the SEM.

Figure 1b provides a scanning electron microscopy image of a Shape 3 toroidal anvil with the torus-depth measurement labeled. Each anvil was milled until the torus depth measured in cross section reached the desired depth given in Table 1 and shown in Fig. 1b. The torus depths were measured in the SEM using a trigonometric relation while the sample stage was tilted at a 52° angle for milling. In each case, the torus depths reported here can be reproduced by measuring the torus-to-culet-bevel distance in cross section intermittently during milling following the example in Fig. 1b. When repeatedly making this measurement on the same anvil, torus depths were measured within an uncertainty of ~ 1 µm. By tracking the milling time per anvil, consistent anvil profiles can then be reproduced setting the milling time with the same milling parameters. As depth measurements in the SEM are challenging due to factors such as choosing the start and end points, correcting for the perspective view, and changes in the anvil surface due to redeposition, we additionally evaluated the dimensions of our tDACs in profile view optically after milling (Fig. S2). For a Shape 3 anvil with a torus depth of ~ 20 µm measured in cross section in the SEM, we measured a ~ 14 µm shoulder depth optically with a microscope with 0.9 µm resolution at the highest magnification (Fig. S2). For a ~ 20 µm torus depth, a Shape 3 anvil should have a 15 µm shoulder depth (Table 1). The uncertainties in depth measurement observed here are small relative to the range of torus depths tested (9–20 µm) and are within the range of anvil torus depths that perform satisfactorily (18–20 µm) as discussed below. To produce the anvil shown in Figure 1b and all anvils shown in Table 1, we also scaled the bitmap to 250 × 250 µm centered on a 200 µm flat anvil prior to milling. The region of the bitmap that overlaps with the anvil pavilion therefore shows a steep drop-off in the milled anvil, providing an additional bevel to the shoulder to counteract anvil deformation under high loading (Fig. 1a,b). The edge of the FIB milled section exhibits a sharp artifact at the intersection of the bitmap slope with the anvil pavilion. By extending the bitmap out onto the pavilion, this sharp edge is also offset significantly lower than the culet such that it does not cause anvil failure, as confirmed by our absorption scans collected under compression (Fig. 1c, Fig. S3). Figure 1a illustrates the starting 200 µm and the idealized bitmap profile, while the resulting profile that includes the artifacts of milling on the pavilion and milling at a high 2.5 µA current in the FIB in general is shown in Fig. 1b. We point out that Shapes 2 and 3 have a stepped shoulder profile in the raw bitmap (Fig. 1a, Fig. S1), but given the scaling of the bitmap onto the pavilion in this region, it is likely that this feature is not effective here but may prove useful if the bitmaps are scaled concentrically with the culet only (Fig. 1a, Fig. S1).

For a 50 µm culet, Shape 1 and 2 profiles with 9–15 um torus depth failed under 2 Mbar due to the shoulders of the anvils deforming under load and cutting through the gasket (Fig. S3a,b). Once the torus depth was increased to 18 µm for a 50 µm culet, the shoulders of the anvils still deformed under compression but were no longer the cause of anvil failure. Instead, the cupping of the culets caused anvil failure still under 2 Mbar (Fig. S3c). A bevel was then introduced to the culets in Shape 3 (Fig. 1a), whereby the 50 µm culet was beveled down to a 30 µm central culet. For this shape, torus depths of 16, 18, and 20 µm were tested (Table 1, Fig. 1). The 16 µm torus depth tested on F001 survived only to ~ 200 GPa, but the 18 µm torus depth and 20 µm torus depth anvils compressed between 249 and 414 GPa (Table 1). It should be noted that C172 was compressed to 249 GPa and held there to test laser heating (Supplemental text S3), and therefore, this pressure is an underestimation of the performance of the anvils in this experiment. The Shape 3 anvils with 20 µm torus depths achieved the highest pressures in this work with half of the tested anvils achieving between 3–4 Mbar (Table 1). A more rigorous evaluation of the pressures obtained and their uncertainties based on the indexed volumes of Pt, Au, and Re are provided for samples C172 and C173 and discussed in the following sections. For C173, the shoulders of the anvils exhibited moderate deformation below 4 Mbar (Fig. 1c), but this deformation did not pinch out the gasket in this compression cycle, and the anvils supported pressures > 4 Mbar. It is likely that the deformation of the culets caused anvil failure at peak pressures (Fig. 1c), as Shape 3 anvils consistently fail via a vertical crack through the culet. We recognize that the performance of these anvils also depends on other factors such as alignment of the anvils and starting sample density, however, these initial tests indicate that the Shape 3 anvils do repeatedly perform to the highest pressures compared to the other tested shapes. A bitmap used to make the Shape 3 anvils is provided (Supplementary Data S1).

At central pressures equal to ~ 3.7 Mbar, determined from Pt^24^, we collected horizontal and vertical X-ray scans of sample C173 to assess the pressure distribution across the anvils. Tracking the Re pressure^25^ across the gasket and sample, pressures increased from ~ 20 to 60 GPa in the shoulder and torus region of the anvils before spiking up to 3.7 Mbar on the 30 µm culets (Fig. S3). The pressure gradient across the culets does not exceed ~ 20 GPa at 3.6 Mbar (Fig. S4). The observed confining pressure, ~ 40–60 GPa, is comparable to that reported for the double-stage diamond anvils^15–18,21^, but less than half that reported for other toroidal diamond anvils^20^.

### Volume and pressure determination

**Figure 2:**
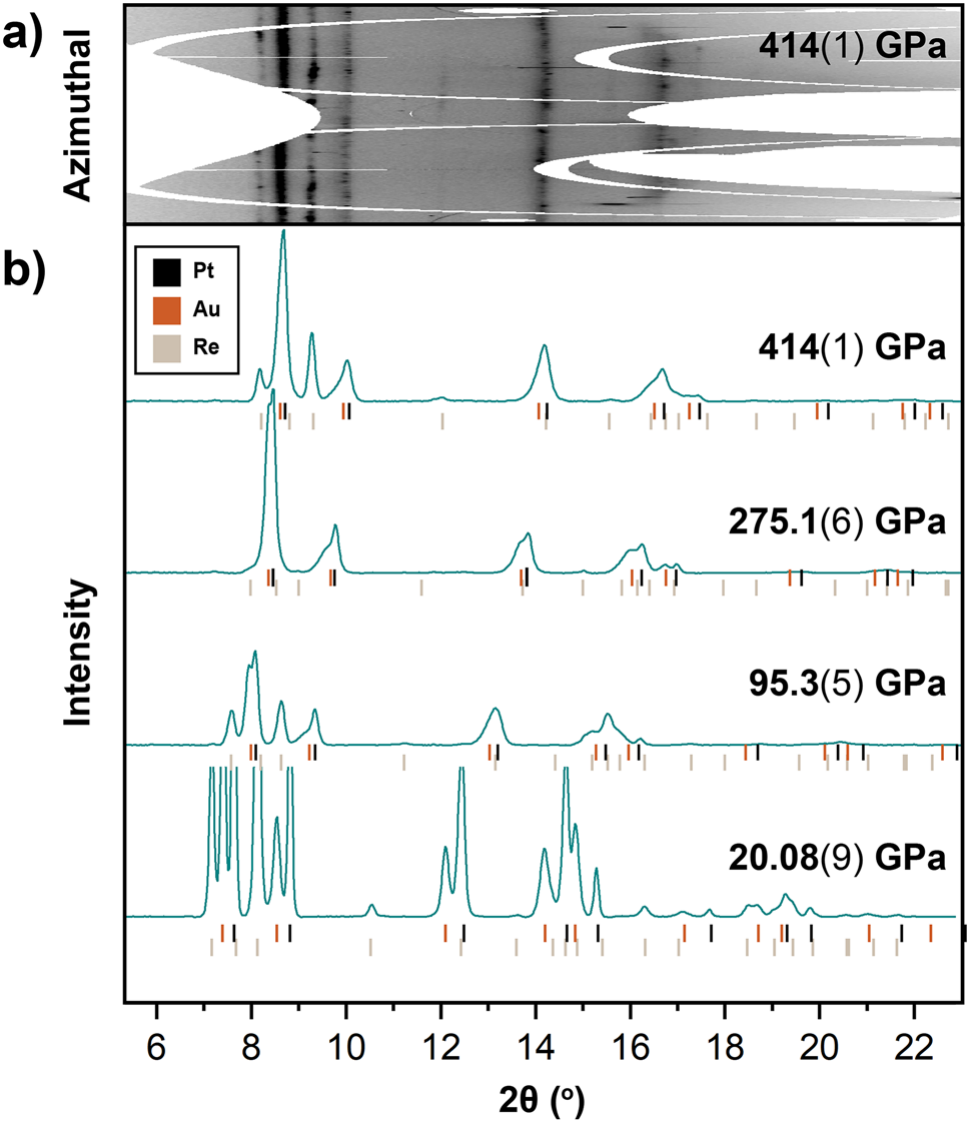
(**a**) X-ray diffraction pattern of sample C173 loaded with Pt and Au at maximum pressures of 414(1) GPa based on the platinum volume calibrated to the ramp compression scale^24^. (**b**) Integrated X-ray diffraction patterns of sample C173 loaded with Pt and Au showing diffraction from Pt, Au, and Re up to 414(1) GPa based on the platinum standard^24^.

**Figure 3:**
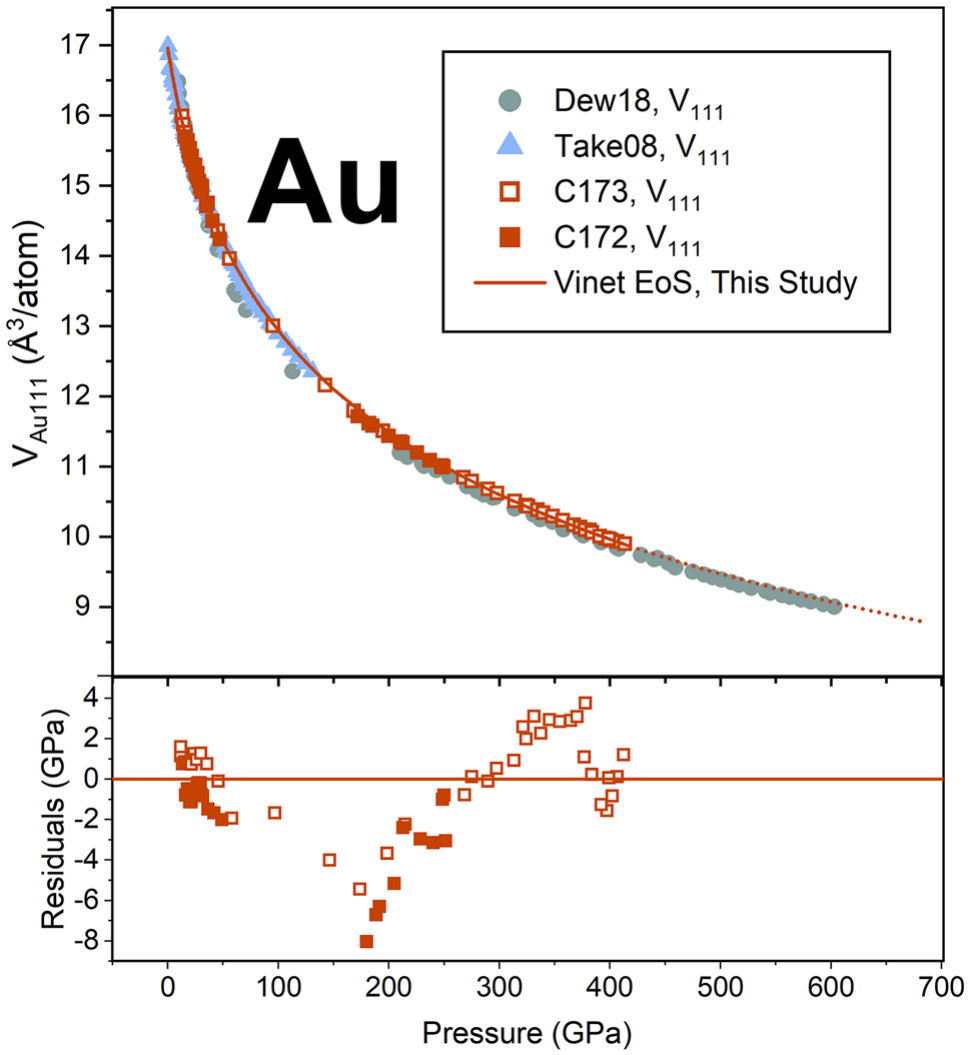
(**a**) Evolution of gold volume calculated from the (111) plane for samples C172 (closed symbols) and C173 (open symbols) run in this study. A Vinet equation of state fit, with *K*_0_ fixed to 167 GPa^32^ and *K*’_0_ fit to 6.075(6) is provided via the orange solid line. Pressure residuals to this EoS fit are included in the bottom panel. Gold volumes calculated from the (111) plane from (19) (green circles) and (26) (blue triangles) are also included for comparison.

The pressure exerted by the toroidal anvils for a nonhydrostatic loading was monitored and validated by measuring the volumes of platinum and gold using synchrotron X-ray diffraction for samples C172 and C173 (Fig. 2). Rhenium was additionally observed from the gasket in sample C173 and its volume was measured (Fig. 2). Volumes were calculated for fcc Pt and Au based on the (111) lattice plane (Fig. 3, Dataset S2). Previous hydrostatic^26^ and non-hydrostatic^19,23^ DAC compression studies have established that the (111) lattice plane of fcc metals including gold and silver is least affected by uniaxial stress while the (200) lattice plane is most affected by uniaxial stress. Therefore, the volume for gold and platinum calculated from the (111) lattice plane is closer to a hydrostatic approximation compared to the volume determined from multiple lattice planes. Additionally, as shown in Fig. 3, above 275 GPa, the fcc Pt and Au peaks overlap to the point where the maxima of Au cannot be differentiated from the higher intensity Pt peak. Once peak overlap initiated, we then held the full-width-half-maximum of the Pt and Au peaks fixed to obtain a consistent peak fitting with further pressurization to 414 GPa. Rhenium volumes were determined from the measured (100), (101), and (102) d-spacings.

We chose Pt as the pressure calibrant, as the volumes indexed from the (111) and (200) lattice planes of Pt do not show any significant deviation with pressure and agree within < 0.7% across this study (Fig. S5, Supplementary dataset S2). As such, the uniaxial stress calculated for Pt based on these volume differences^27^ do not show a trend with pressure and reach at most 1.6 GPa across this study (Fig. S5, Supplementary text S2). These results suggest that Pt compresses relatively isotropically and does not maintain significant uniaxial stress, despite the nonhydrostatic compression environment and is a reliable ultra-high-pressure standard. For pressure calibration, the volume obtained from the (111) peak of platinum was considered, as it is the closest approximation to the hydrostatic volume based studies of analogous fcc metals^19,23,26^. We chose to calibrate our platinum volumes to the recent ramp compression Pt scale^24^, as this scale is calibrated beyond the pressures of this study and is compatible with previous DAC studies^28–30^ (Fig. S6). For samples C172 and C173, the application of the Pt pressure scale^24^ results in a maximum obtained pressure of 249.2(3) GPa and 414(1) GPa, respectively.

### Equation of state of gold to 4.1 Mbar

Figure 3a shows the evolution of gold volumes indexed from the (111) lattice plane with platinum pressure compared to the gold volumes indexed from the (111) lattice plane reported by (19) (nonhydrostatic) and (28) (helium pressure-transmitting medium). The gold volumes indexed for samples C172 and C173 show a smoothly decreasing trend with pressure and are in agreement with each other and with previous hydrostatic compression studies^26^. Our indexed volumes are marginally larger than that reported by (19), which may reflect that our compression conditions were less hydrostatic or it may reflect differences in the pressure calibrants used.

We then fit a Vinet equation of state to our Au (111) volumes following the form:

1 $$P = 3K_{0} \left\{ {[1 - (V/V_{0} )^{1/3} ]/(V/V_{0} )^{2/3} } \right\}e^{{3/2(K^{\prime} - 1)(1 - (V/V0)^{1/3} )}} ,$$

**Figure 4:**
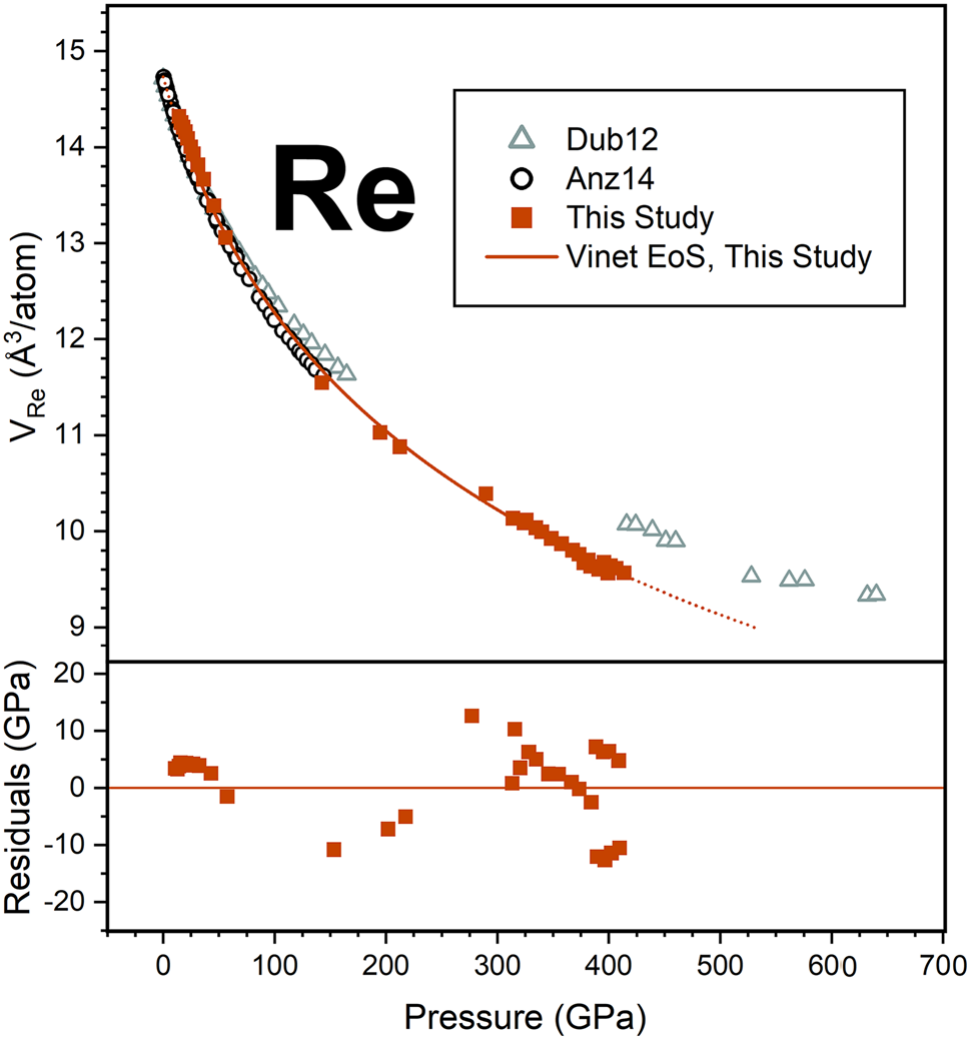
Rhenium volumes calculated from the measured (100), (101), and (102) lattice spacings calibrated to the platinum standard^24^ for this study (orange squares). The Vinet equation of state fit to our data with *K*_0_ fixed to 360 GPa^33^ and *K’*_0_ fit to 4.52(3) is provided by the solid orange line. Pressure residuals to this fit are included in the bottom panel. For comparison, rhenium volumes reported by^25^, calibrated to ruby^42^, tungsten^42^, and helium^43^ pressure standards are included (black open circles), and rhenium volumes reported by^13^ calibrated to gold^33^ are also included (grey open triangles).

We provide several fittings in Table 2. Considering the merging of the Au and Pt peaks with pressure, where the Au (111) peak maxima cannot be independently observed above 275 GPa (Fig. 2), we fit equations of state to our data up to 275 GPa as well as up to 414 GPa for comparison. In all scenarios, we fixed the ambient volume to 16.962 Å^3^ from^31^. We report fits with the ambient isothermal bulk modulus (*K*_0_) fixed to the ultra-sonically derived value of 167 GPa^32^ and as a free parameter (Table 2). Despite the peak overlap observed in Au and Pt at the conditions reached in this study, comparison of the EoS fits with *V*_0_ = 16.962 Å^3^ and *K*_0_ = 167 GPa up to 275 GPa and 414 GPa reveals that the fitted *K*’ equals 6.05(1) and 6.075(6), respectively (Table 2). These results support that even adopting a conservative equation of state fit to the non-overlapping data, our fitted volumes in the 275–414 GPa range are still reasonably described by these fitting parameters. Figure 4 includes the equation of state fit to our gold V_111_ volumes up to 414 GPa with *V*_0_ = 16.962 Å^3^, *K*_0_ = 167 GPa, and *K*’_0_ = 6.075(6). Pressure residuals to this fit reach at most a 4% deviation from the reported Pt pressure (Fig. 4). The *K*_0_’ value for this nonhydrostatic fit is 3% larger than *K*_0_’ value reported in the hydrostatic compression study (26). Equation of state fits for Au from previous studies^19,24,26,30,33–35^ are also included in Table 2 for comparison.

**Table 2:** Vinet equation of state parameters for gold.

Au EoS	Method	Fit to	P calibration	PTM	P_max_	V0	K0	K'	Note
					GPa	Å^3^/at	GPa		
This study	DAC, XRD	(111)	Pt, Frat21	None	414 (1)	16.962*^, a^	167*^, b^	6.075 (6)	
This study	DAC, XRD	(111)	Pt, Frat21	None	414 (1)	16.962*^, a^	157 (2)	6.35 (4)	
This study	DAC, XRD	(111)	Pt, Frat21	None	275.1 (6)	16.962*^, a^	167*^, b^	6.05 (1)	
This study	DAC, XRD	(111)	Pt, Frat21	None	275.1 (6)	16.962*^, a^	163 (2)	6.18 (5)	
Dewaele18	DAC, XRD	(111)	Re, Anz14	None	603	16.962	167*^, a^	5.94 (1)	
Takemura08	DAC, XRD	(111)	Ruby, Zha00	He	131	16.962	167*^, a^	5.9 (1)	
Fratanduono21	Ramp	–	–	–	1000	16.929	170.90 (24)	5.880 (5)	
Dorfman12	DAC, XRD	(111), (200), (220)	MgO, Tan09	Ne	253	16.962	167*^, a^	5.88 (2)	
Yokoo09	Shock	–	–	–	550	16.929	167.5	5.94	
Fei07	DAC, XRD	varies between combined studies	Ruby, Dew04; MgO, Speziale01	Ne, He, NaCl	103	16.929	167*^, a^	6.00 (2)	Combined Fei07, Dew04, Hirose06 data
Golding	Ultrasonic	–	–	–	0.10	–	166.7	6.21	

*Parameters fixed during fit.

^a^V_0_ from^31^.

^b^K_0_ from ultrasonic data^32^.

### Equation of state of rhenium to 4.1 Mbar

In Experiment C173, the volumes of rhenium under nonhydrostatic loading conditions from the gasket were also determined up to 414(1) GPa from the measured (100), (101), and (102) lattice spacings. Rhenium volumes show a smoothly decreasing trend with pressure and are calibrated to the Pt pressure scale^24^ (Fig. 4). Comparisons to previous *P–V* relationships of rhenium up to multi-megabar pressures reveal that the compression data determined in this study are compatible with the compression curve of rhenium in helium reported by^25^, and not compatible with the *P–V* relationship reported via double stage DAC compression experiments in He and Ne by^13^ (Fig. 4). A Vinet equation of state was fit to the data assuming an ambient volume of 14.733 Å^3^ per atom after^25^ and fixing the ambient bulk modulus to the ultra-sonic value of 360 GPa^36^. As all tDAC samples started at some non-zero pressure, we assumed an ambient volume following^25^. This fit is shown in Fig. 4, and fits reported by additional studies^13,25,36–41^ are provided for further comparison in Table 3. We conducted an EoS fit to our Re data allowing both *K*_0_ and *K′* as fitting parameters as well and listed these results in Table 3.

**Table 3:** Equation of state parameters for rhenium.

Re EoS	Method	P calibration	PTM	P_max_	V0	K0	K'	K”	Fit
				GPa	Å^3^/at	GPa			
This study	DAC	Pt (Frat21)	None	414 (1)	14.733*^, a^	360*^, b^	4.73 (2)		Vinet
This study	DAC	Pt (Frat21)	None	414 (1)	14.733*^, a^	374 (17)	4.5 (2)		Vinet
Anzellini14	DAC	Ruby (Doro07), W (Doro07), He (Loub93)	He	144	14.733	352.6	4.56		Vinet
Dubrov12	DAC	Au (Yok09)	He	640	14.728	348	7.57^c^		Vinet
Dubrov12	DAC	Au (Yok09)	He	640	14.728	348	6.07^d^		Vinet
Liu70	DAC	NaCl	NaCl	35	14.712	336	4*		BM2
Vohra87	DAC	Ruby	–	216	14.715	372	4.05		BM3
Jeanloz91	DAC	Ruby	4:1, none	170	–	360.3	5.43	-0.06	BM4
Lv12	–	–	–	–	14.673	376	4.58		GGA-PBE
Sikka88	–	–	–	–	–	372	5.41		LMTO
Manghnani74	Ultrasonic	–	–	0.42	–	360.3	5.43		–

*Parameters fixed during fit^a^ V_0_ fixed to value reported from X-ray data by^25^.

^a^K_0_ fixed to value reported from ultrasonic data by^36^.

^b^Fit parameter reported by^13^.

^c^Fit parameter refit by^25^.

## Discussion

### Toroidal anvil performance comparison

The performance of the anvils tested in the current study is compared to the pressure-loading curves of other toroidal characterizations^19,20^ and standard anvil characterizations^44^ in Fig. 4. Standard, bevel, and toroidal anvils all undergo a similar compression path with increasing force on the cell (Fig. 4). Initially, in Stage 1, pressure on the sample linearly increases as the sample material is compacting, then in Stage 2, the sample experiences a more rapid increase in pressure as it is fully compacted and all force is going into elastic deformation of the anvils^19^. For all anvil types, Stage 1 compression is observed to < 50 GPa (Fig. 4). For a 20 μm standard beveled anvil, the Stage 2 increase in pressure extends to ~ 210 GPa^44^. By comparison, the toroidal anvils undergo Stage 2 compression up to ~ 280 GPa for the current study, ~ 220 GPa for^19^, and ~ 180 GPa for^20^. Above these loads, Stage 3 occurs when the diamonds have reached a strain limit on the culets and cupping of the culets for the standard beveled anvils and cupping of the shoulders for the toroidal anvils continues until anvil failure^19,20,44^. Interestingly, in this stage, our anvils behave more like a standard bevel than the toroidal anvils, where the cupping of the culet is the driving cause of failure in Stage 3 compression (Fig. 1c). Routinely, our anvils extend to the highest pressures in the Stage 2 compression (Fig. 4), while the confining pressure on our anvils in the Stage 3 compression regime ~ 3 × lower than that reported for other toroidal anvils^20^. Recent work^21^ exploring the effects of increasing confining pressure on the maximum attainable sample pressure, indicates a positive correlation, which suggests that further modifications to the torus and shoulder of our anvil profiles to increase the confining pressure may help to achieve even higher pressures and greater stability for our large volume anvils in Stages 2 and 3.

**Figure 5:**
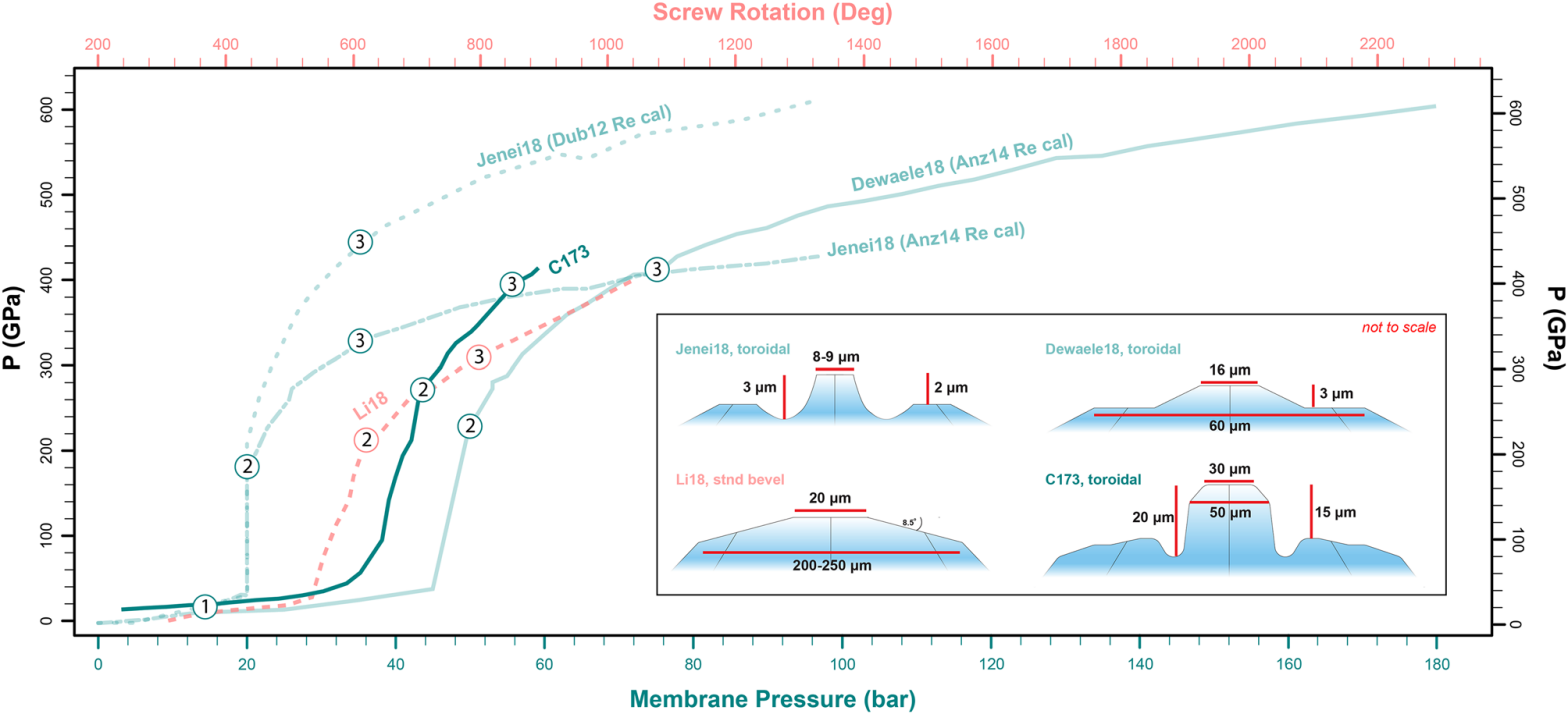
Cell pressure with membrane pressure (bar) or screw rotation (deg) for the highest-pressure cell run in this study (C173) compared to other toroidal anvil and standard bevel anvil studies^19,20,45^. The colors of the lines and data are associated with the relevant x-axes, depending on whether the study reported the loading curve from membrane compression or manual screw turning. The shapes of the anvils associated with each pressure-loading curve are provided in the inset. Two curves are presented for^20^, one with the Re pressure calibrated to^13^ and the other with the Re pressure calibrated to^35^.

As our rhenium *P–V* data shows compatibility with^25^, we can compare the behavior of our toroidal anvils under membrane loading with the toroidal anvil pressure-loading curves^19,20^ also calibrated to^35^ (Fig. 5). For this comparison, we therefore recalibrated the membrane compression curve for the V3-2 anvils reported by^20^ from the Re scale by^13^ to the Re scale by^25^. The membrane compression curve for^20^ calibrated to both scales^13,25^ are shown in Fig. 4. In this context, our toroidal anvils achieved a similar maximum pressure to that reported by^20^, even though the optimized toroidal profile presented here has a culet that is 3.75 times larger in diameter compared to the profile presented^20^. If we estimate that the samples contained in these anvils are cylindrical with the same thickness, our anvils can support a sample that is 3.75 × wider and therefore 14 × larger volumes compared ^20^. Additionally, our toroidal anvil configuration out-performs even smaller culet conventional beveled diamond anvils^44^ (Fig. 5). The maximum pressures reached in this study for a 30 µm culet trend above the pressure routinely achieved with the same culet diameter for standard beveled anvils^44.^ While 300 GPa is nominally the reported maximum pressure for this culet diameter in the literature^45^, the toroidal anvils that achieved > 300 GPa presented here highlight that Stage 2 compression of these anvils typically occurs between 50 and 280 GPa followed by the Stage 3 compression up to 4 Mbar. Given the asymptotic slope of membrane compression with sample pressure in Stage 2 compression (Fig. 5), the 30 µm toroidal anvils presented in this study compress efficiently to ~ 3 Mbar once in the Stage 2 regime, while standard anvils of even smaller culet diameter compress efficiently to ~ 2.2 Mbar then require more linear, Stage 3 compression to achieve 3 Mbar. This is important to consider, as the current anvils offer access into the 3–4 Mbar regime with 30 µm culets, with the caveat of lower data density in the 50–280 GPa range given the rapid rise of sample pressure with little force in Stage 2 compression. There is still room for refinement of these larger-volume toroidal anvils, but the optimized shape presented here offers a strong basis for reaching ultrahigh pressures with sample volumes suitable for complex sample configurations. This includes, embedding the sample in a soft medium to improve the compression environment in the chamber, loading the sample embedded in thermal insulation for laser heating, and loading multi-layer samples to react together at high pressures and temperatures. Supplemental text S3 provides additional discussion for using these anvils under high pressure–temperature conditions.

### Equation of state comparisons for gold and platinum

**Figure 6:**
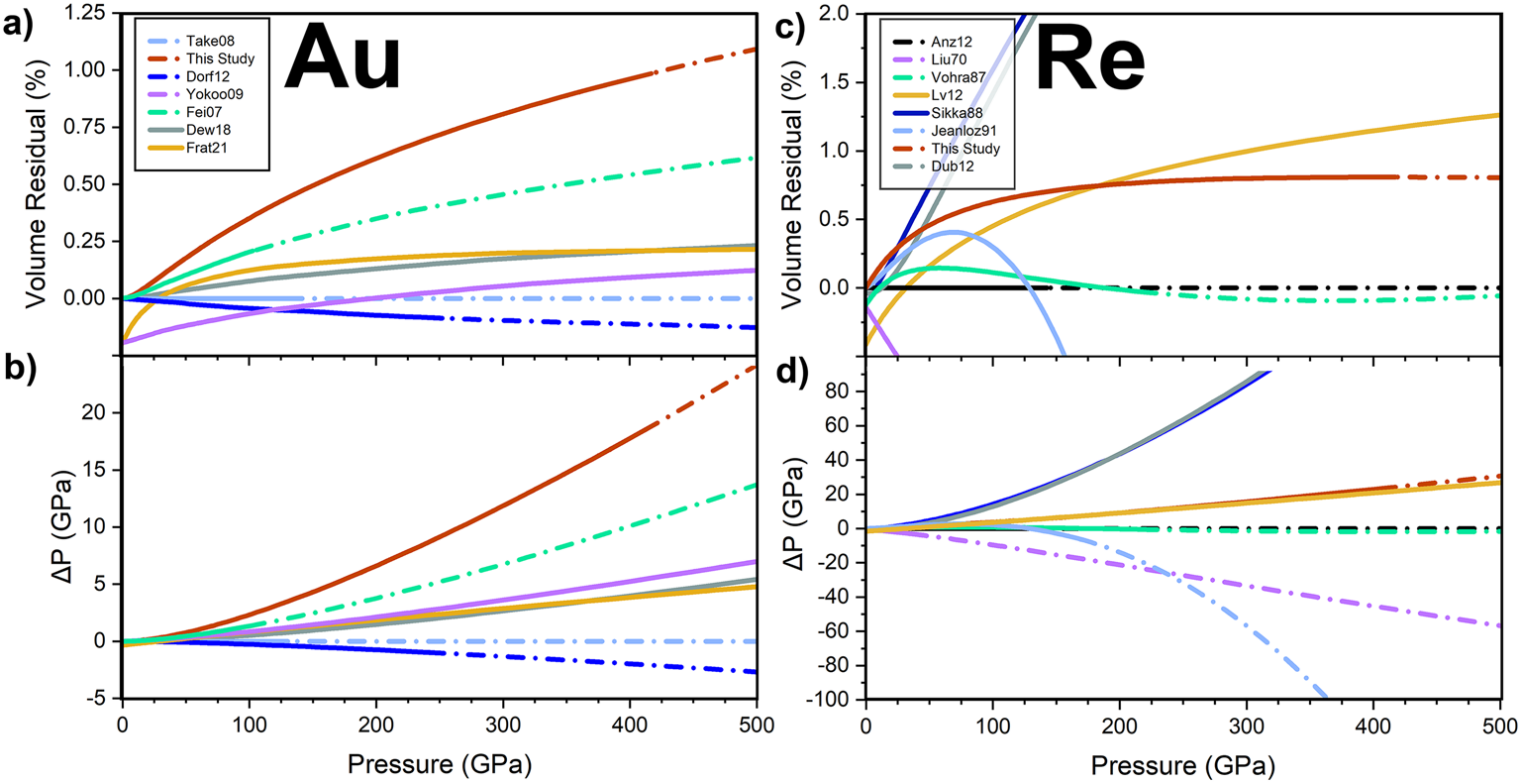
(**a**) Percent volume and (**b**) pressure residuals of the equations of state fits from this study (*V*_111_)^19,24,30,33,34^. (**a**) compared to the equation of state fit to gold in helium^26^. (**c**) Percent volume  and (**d**) pressure residuals of the rhenium equation of state fits from this study^13^, and ^37–41^compared to the equation of state fit to rhenium in helium^25^. The solid lines represent the data coverage of each study, and the dashed lines represent the extrapolations of the fits.

The gold and rhenium data collected in this study provide an independent check and evaluation of the Au and Re pressure scales to > 4 Mbar. Our results for the compression of gold under nonhydrostatic conditions in the toroidal diamond anvil cell agree with a multitude of previous static and dynamic equations of state of gold within ~ 1% by volume up to 4 Mbar (Fig. 6a)^19,24,26,30,33,34^. This volume difference would result in a pressure difference of ~ 20 GPa at 4 Mbar (~5 %) (Fig. 6b).

The equation of state fit to the rhenium volumes determined in this study is within ~ 0.75% volume and ~ 20 GPa of the extrapolated equation of state of rhenium in helium^35^ to 4 Mbar (Fig. 6c,d). Compared to other Re equation of state studies^13,37–41^, our results are most similar with the first principles EoS by^41^. Alternatively, the equations of state determined experimentally by^13^ and theoretically by^39^ reports ~ 100 GPa higher pressures than the hydrostatic extrapolation^25^ by 320 GPa (Fig. 6). The equations of state reported by^37,40^ diverge significantly in the negative direction above ~ 130 GPa from the hydrostatic extrapolation^25^. The results from this study indicate that under nonhydrostatic loading conditions up to > 4 Mbar, rhenium in our experiments behaves more similarly to^25^ compared to these previous experimental and computational reports. Results from this study help to validate rhenium under nonhydrostatic compression as a pressure calibrant by providing data in support of recent works using this pressure scale^25^ to 6 Mbar^19^.

Unifying these *P–V* relationships to a single pressure scale will further improve comparisons of the gold and rhenium equations of state discussed here, but agreement between our data and extrapolations of equations of state of gold and rhenium in helium^25,26^, validate the performance of the large-volume toroidal anvils tested here to > 4 Mbar. With further tests of laser heating using such large-volume toroidal anvils, we are close to generating *P–V–T* data of key pressure standards over a wider pressure–temperature range that would lead to an internally consistent pressure scale for planetary materials applications.

## Conclusion

In this study, we aimed to present a toroidal anvil profile that optimizes sample volume for compression studies above 300 GPa. Increasing sample size aids in reaching ultrahigh pressure-temperature conditions where many exotic physics and chemistry phenomena have been predicted and that are relevant to Earth and exoplanetary interiors. We explored the relationship between culet diameter, torus depth, and shoulder depth for anvils with central culets in the 30–50 µm range. Using toroidal anvils with 30 µm culets, torus depths of 20 µm and shoulder depths of 15 µm, we achieved a maximum pressure of 414(1) GPa based on the platinum pressure scale. Equations of state fitted to our gold (111) and rhenium volumes agree within 1% and 0.75% by volume of previously reported hydrostatic equations of state for these materials extrapolated to 4 Mbar, respectively. These results cross-validate that the toroidal anvils tested in this study reached > 4 Mbar and provide a promising basis to further develop these large-volume toroidal anvils for exploring exotic materials and planetary petrology at high temperatures in the 3–4 Mbar range.

## Methods

### Anvil fabrication

Toroidal diamond anvils were fabricated at the Carnegie Institution for Science using an FEI Helios plasma focused ion beam (PFIB) G4 with a xenon source. Type Ia, standard-cut 200 µm culet diamond anvils sourced from Almax easyLab were used as the base for toroidal milling. The toroidal profiles were imported as bitmaps into the PFIB and scaled to a 250 µm by 250 µm milling region centered on the culet. Anvil milling was performed using an ion energy of 30 kV and beam current of 2.5 µA. As the FIB milling depth for this instrument is calibrated to silicon, the milling process for each anvil was monitored manually by intermittently pausing the milling and measuring the inner culet height using the “cross-section” setting. The anvils were considered complete when the desired torus depth was achieved within ~ 1 µm. The torus depth was repeatedly measured as shown in Fig. 1b, from the base of the torus where redeposition on the walls of the culet is observed to the edge of the culet bevel where the redeposition from anvil milling ends. Additionally, all anvils were milled radially outwards to reduce redeposition on the anvil tip. Total milling time typically ranged from 1 to 1.5 h per diamond. The bitmap used for the highest-pressure experiments reported here is included in the supplementary material (Supplementary file S1).

### Sample preparation

The milled toroidal diamonds were mounted on tungsten carbide seats. Sample chambers were prepared by indenting rhenium or tungsten gaskets to 12–15 µm thick, drilling an 8–12 µm sample chamber, and filling the sample chamber with pressed foils of platinum and gold as pressure markers for X-ray diffraction. Select samples were also loaded with SiO_2_ to test multi-layer sample configurations for future laser heating as well as cBN inserts to increase sample thickness and reduce the diffraction signal from the gasket (Table 1).

### Anvil performance evaluation using X-ray diffraction

The performance of the toroidal anvils was monitored using angle-dispersive synchrotron X-ray diffraction at Argonne National Laboratory, Sector 13 ID-D of the Advanced Photon Source. An X-ray beam measured at 2.5 µm × 3.54 µm full width, half maximum tuned to 42 keV was used to probe the sample chamber under compression. X-ray diffraction was collected on a CdTe 1 M Pilatus detector calibrated using a LaB_6_ NIST powder standard. Upon compression, X-ray absorption scans of the milled region of the anvils were collected to monitor the deformation of the anvils with pressure. The pressure generated by the anvils was determined by collecting X-ray diffraction from platinum, gold, and rhenium on the central culet. X-ray exposure times of 1–2 s were used. Diffraction data was monitored during experiments using Dioptas^46^ and processed using in-house OriginC codes. Pressure was calibrated using the equation of state of platinum^24^ for measurements in the sample chamber and of rhenium^25^ for measurements collected scanning across the gasket.

### High temperature testing

On select samples, X-ray diffraction was collected during laser heating. Samples were heated with 100 W infrared fiber lasers in double sided configuration at Sector 13 ID-D, APS. The laser beam was shaped to a10 µm flat-top and aligned to the X-ray beam using the fluorescence of the sample^47^. The laser shutter was incrementally opened on the sample at a set laser power and exposure in the “burst” heating mode. The thermal emission collected from the central 6 µm of the laser flat top was recorded using an Action SP-2360 imaging spectrograph with a PI-MAX3 1024i ICCD camera (Princeton Instruments). Temperatures were determined by fitting the thermal emission profile to a grey-body approximation^48^.

### Supplementary Information

Supplementary Information 1.

Supplementary Information 2.

## Data Availability

The data used to obtain the results and conclusions presented in this work are provided in the main and supplemental texts S1 as well in Supplemental Datasets S1, S2 and S3. Additional data related to this paper may be provided upon request to the corresponding authors.
