# Supplementary material for: Exploring toroidal anvil profiles for larger sample volumes above 4 Mbar
Source: Sci Rep. 2024 May 18;14:11412. doi: 10.1038/s41598-024-61861-2 (PMC11102561; doi:10.1038/s41598-024-61861-2)
Supplement: Supplementary file 1 — Supplementary Information 1. [file 41598_2024_61861_MOESM1_ESM.docx]

**Supplemental information for:**

**“Exploring toroidal anvil profiles for larger sample volumes above 4 Mbar”**

**Claire C. Zurkowski*^,a,b,¥^, Jing Yang^a^, Francesca Miozzi^a^, Suzy Vitale^a^, E. F. O’Bannon III^b^, Zsolt. Jenei^b^, Stella Chariton^c^, Vitali Prakapenka^c^, Yingwei Fei*^,a^**

^a^Earth and Planets Laboratory, Carnegie Institution for Science, 5241 Broad Branch Road, NW, Washington, DC 20015, USA

^b^Lawrence Livermore National Laboratory, 7000 East Ave, Livermore CA 94600, USA

^c^Center for Advanced Radiation Sources, The University of Chicago, 9700 South Cass Avenue, Building 434A, Argonne, IL 60439, USA

^¥^Now at, Lawrence Livermore National Laboratory, 7000 East Ave, Livermore CA 94600, USA

Corresponding Authors: Claire Zurkowski, [zurkowski1@llnl.gov](mailto:zurkowski1@llnl.gov); Yingwei Fei, yfei@carnegiescience.edu

**Table of Contents:**

1. **Supplementary datasets S1:** Bitmap of Shape 3
2. **Supplementary datasets S2:** Pt, Au, Re d-spacings for C172 and C173
3. **Supplementary text S1:** Extended discussion of anvil testing
4. **Supplementary text S2:** Extended discussion on the uniaxial stress analysis of Pt
5. **Supplementary text S3:** Extended discussion on the use of these anvils under high pressure and high temperature conditions
6. **Figure S1:** Schematic of all anvil designs tested
7. **Figure S2:** Comparison of the tDAC specifications with the SEM vs. optical microscope
8. **Figure S3:** X-ray transmission scans of Shapes 1 and 2 tested to 2 Mbar
9. **Figure S4:** Confining pressure measured on C173 at 367 GPa
10. **Figure S5:** Percent volume residuals of the Pt the (200) lattice spacing compared to the Pt (111) lattice spacing and uniaxial stress of Pt determined from the (111) and (200) lattice spacings
11. **Figure S6:** Comparisons of previous DAC and ramp compression Pt scales

**Supplementary text S1.**

**Extended discussion of anvil testing**

The first toroidal profile tested consisted of a 50 µm culet, 9 µm torus depth, and 6 µm shoulder depth (Figure S2a). At moderate pressures, the X-ray absorption scans of the anvils indicate that the shoulders significantly cupped upwards. The diamonds pulverized at 165 GPa because the shoulders of the anvils punched through the gasket (Figure 2a). The next tests then incorporated a deeper torus and shoulder and a stepped shoulder edge to counter diamond cupping under compression. With 12-15 µm torus depths and 50 µm culets, all the diamonds failed at pressures below 200 GPa and exhibit significant cupping that led to anvil failure. For sample C161, consisting of Shape 2 anvils with a 15-um torus depth and 11.25 µm shoulder depth, the deformation of the shoulders was still the major issue that resulted in failure of the diamonds (Figure S2b). Additionally, the culets themselves cupped under moderate pressures (Figure S2b). These initial tests indicated that for these large culets, whether the shoulder depth was 2/3 or ¾ the torus depth (Shape 1 vs. Shape 2), all anvils with torus depth less than 15 µm exhibited significant shoulder deformation that led to failure below 200 GPa.

The next toroidal tests maintained a shoulder depth of ¾ the torus depth and continued to increase the torus depth greater than 15 µm to counteract anvil cupping. Once the torus depth was increased to 18 µm for a 50 µm culet, the shoulders of anvils finally maintained their shape under pressure and showed little deformation with increasing pressure (Figure S2c). For sample C164, the deformation of the anvil shoulders did not significantly change above ~75 GPa and the mode of failure of the diamonds was a vertical crack through the culet, suggesting that the anvil had reached a maximum load (Figure S2c). The culets also exhibited cupping during this compression (Figure S2c).

With further toroidal tests, the ratio of shoulder depth to torus depth was maintained and torus depths > 15 µm were pursued. To increase the maximum pressure exerted by the anvils for the same load, we then focused on changing the profile of the central culet (Figure 1a). Additionally, adjusting the culet shape also served to counteract the cupping of the culets observed in earlier in previous runs (Figure S2). A bevel was introduced to the culets in Shape 3, whereby the 50 µm culet was beveled down to a 30 µm central culet (Figure 1a). For this shape, torus depths of 16, 18, and 20 µm were tested (Table 1). The 16 µm torus depth tested on F001 survived only to 200 GPa, but the 18 µm torus depth and 20 µm torus depth samples compressed to 270 GPa and 409 GPa, respectively. It should be noted that for sample C172 with the 18 µm torus depth, we stopped compressing at ~250 GPa to test heating, so this is likely not reflective of the maximum pressure that these anvils may have reached prior to failure. For C173 even at the failure point of 409 GPa, the shoulders of the anvils exhibited only moderate deformation below 4 Mbar (Figure 1c). The failure of these anvils was not due to shoulder deformation but likely due to culet deformation (Figure 1c, d).

**Supplementary text S2.**

**Extended discussion on the uniaxial stress analysis of Pt**

We quantified the uniaxial stress experienced by the platinum sample in this study using the differences in d-spacings measured for the (111) and (200) lattice planes of platinum following the uniaxial stress model^1^ for materials under nonhydrostatic compression. For a cubic system, the indexed nonhydrostatic lattice parameter, *a* determined from a given (*hkl*) can be written as:

1. *a_hkl_* = *M*_0_ *+ M*_1_[3Γ(1 – 3sin^2^*θ*)]

with

1. *M*_0_ = *a*_p_{[1 + (α*t*/3)(1 − 3sin^2^θ ) × (*S*_11_ − *S*_12_ ) − (1 − α^−1^ )(2*G_V_* )^−1^ ]},
2. *M*_1_ = − *a*_p_(α*St*/3),
3. Γ*_hkl_* _=_ (*h*^2^*k*^2^ + *k*^2^*l*^2^ + *h*^2^*l*^2^ )/(*h*^2^ + *k*^2^ + *l*^2^)^2^,
4. *S* = *S*_11_ - *S*_12_ - *S*_44_/2.

The variable *a*_p_ represents the corrected hydrostatic lattice parameter that we aim to determine. Theta (*θ*) is the ½ of the Bragg scattering angle measured for the given lattice plane. The *S_ij_* parameters are the single-crystal elastic compliance components that are inversely related to the elastic constants. Here, the elastic constants and their pressure derivatives from (1) were used: *C*_11_^0^ = 415.09 GPa, *C*_12_^0^ = 297.68 GPa, and *C*_44_^0^ = 91.818 GPa; *dC*_11_/*dP* = 4.335, *dC*_12_/*dP* = 3.6015, *dC*_44_/*dP* = 1.2782. The α term is a correction factor in the range of 0.5 - 1 that determines the stress of the sample. We applied the most conservative lattice parameter correction with α = 1, such that the equation for the hydrostatic lattice parameter, *a_p_* becomes:

1. *a*_p_ = *a_hkl_* / {1 + (*t*/3)(1 – 3sin^2^*θ*)[*S*_11_ – *S*_12_ – 3*S* Γ*_hkl_*]}.

The final variable that needs to be determined in this equation is *t*— the uniaxial stress defined as the difference in stress experienced by the sample in the radial and axial directions. Positive uniaxial stress values represent higher stress along the axial direction. The uniaxial stress can be calculated at every pressure point by finding the slope, *M*_1_, and intercept, *M*_0_, to equation (1), and solving for *t* using equation (3). Previous studies establishing^1^ and applying^3^ this model have approximated that *M*_0_ ~ *a*_p_ for the *t* calculation, such that with α = 1, *t* can be approximated as

1. *t* = -3*M*_1_/(*SM_0_*).

Figure S4 shows the *t* values for platinum calculated for this study based on the reported d_111_ and d_200_ values.

**Supplementary text S3.**

**Extended discussion on the use of these anvils under high pressure and high temperature conditions**

Using these anvils, we have tested 3–4-layer sample assemblies including Pt and Au embedded in MgO, SiO_2_, and KCl insulation and have conducted initial laser heating tests. The use of these transparent pressure media was insightful to the behavior of these anvils. A major challenge of toroidal experiments in general that are made obvious by the incorporation of a transparent pressure medium, is that the sample chamber consistently expands to the center of the torus at some point during the compression or during heating. During compression, it presumably occurs if the sample hole was overfilled and during heating it occurs if the thin gaskets it heated by the laser beam. Our initial heating tests suggest that short duration heating is critical to heat these very thin samples without transferring significant heat to the gasket. Future work will continue to explore the high temperature aspect of using these anvils.

In general, gasket thinning, and material outflow is commonly observed especially in the Stage II region of the compression where the pressure in the sample chamber is rapidly changing. With this, the diffraction intensity from the sample is greatly diminished and is exacerbated by the uniaxial strain on the crystals in the sample. In our heating tests, we did promisingly observe that samples can recrystallize grains of high intensity despite the thinness of the sample. With this, going forward, these anvils may be best used to explore high strength and high *Z* materials above 300 GPa, as they will maintain their structure and diffraction intensity even under nonhydrostatic stress conditions. For planetary materials, it will be important to work towards gas loading samples in these toroidal anvils, or towards laser annealing at incremental pressure steps. It will be challenging, however, to do any laser annealing during the Stage II compression where the pressure in the sample chamber is rapidly increasing. In the context of data that currently exists in the high-pressure community, these anvils are not likely to provide better quality data than what exists below 300 GPa, but once at 300 GPa and entering the Stage III compression, these anvils offer opportunities to develop methods for collecting *P-V-T* data in the 300-400 GPa range.


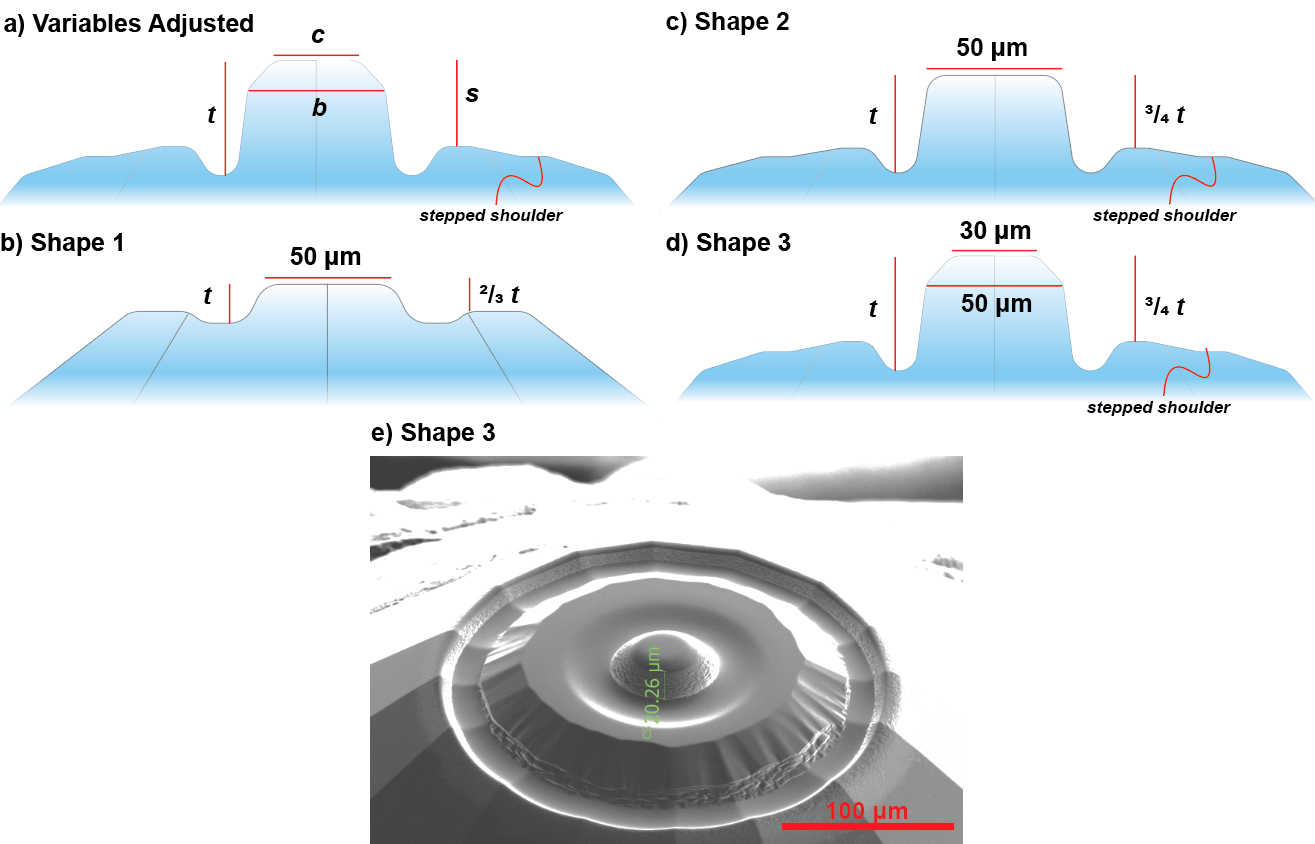


**Figure S1.** a) General schematic of variables adjusted in the milled toroidal anvil shapes tested in this study. To enhance the performance of the toroidal anvils and achieve increasingly higher pressures, the culet diameter (*c*), bevel diameter (*b*), torus depth (*t*), and shoulder depth (*s*) were adjusted in shapes 1, 2 and 3 (b-d). e) Example of the toroidal anvil shape 3 milled with the focused ion beam. Since the milling region measures 250 x 250 µm and the starting anvils are 200 µm in diameter, the outer region of the shoulder exhibits a more rapid gradient where the bitmap is milling on the diamond pavilion. The outer sharp lip of the anvils is an artifact of the differing slopes between the anvil pavilion and bitmap, but this was not observed to be the cause of diamond failure in any experiment, as shown in the absorption scans in Figure 1 and Figure S2. The stepped shoulder added to Shapes 2 and 3 overlapped with the milling region on the pavilion was therefore inconsequential to the performance of these anvils, but this feature may aid in anvil performance for bitmaps maintained within the culet diameter.


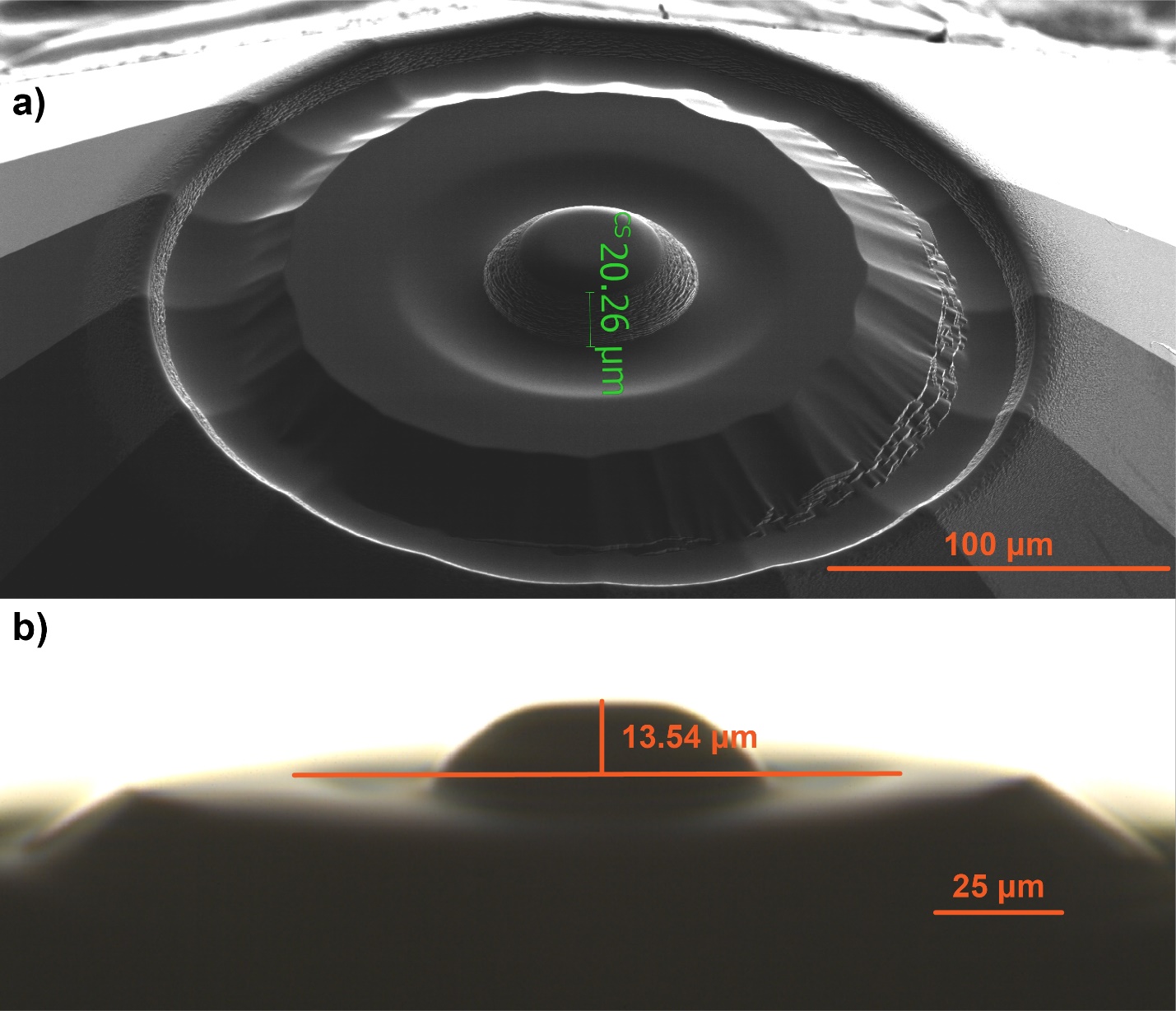


**Figure S2. a)** Example of the toroidal anvil shape 3 milled with the focused ion beam with a ~20 µm torus depth measured in cross section. **b)** Optical view of this shape-3 anvil in profile with the shoulder depth measured to ~14 µm. For this anvil design with a 20 µm torus depth, a 15 µm shoulder depth is anticipated, and compatible results were measured.


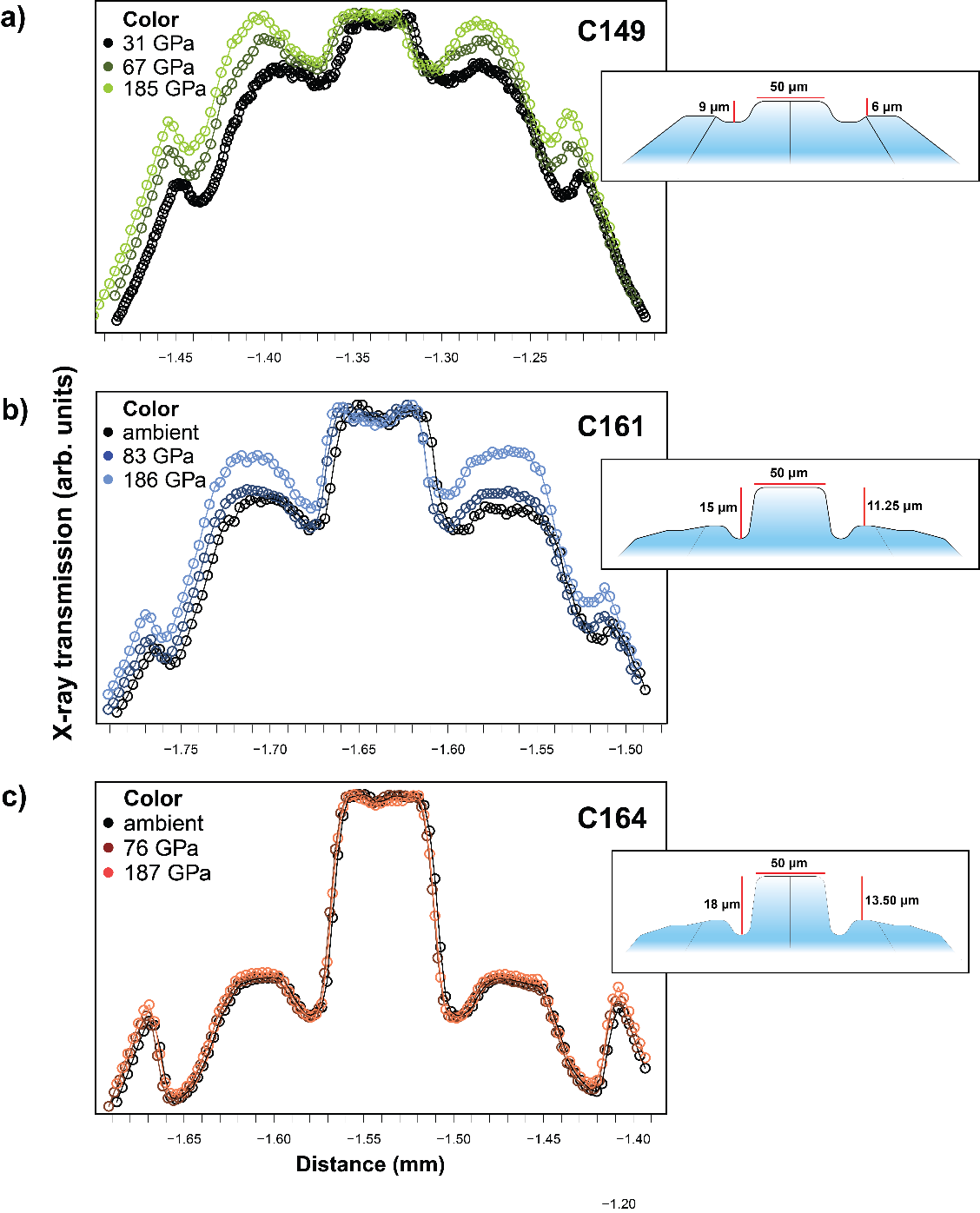


**Figure S3**. X-ray absorption scans collected with increasing pressure on the various toroidal anvil shapes. a) For C149 with a Shape 1 profile and 9 µm torus depth, the anvil shoulders experienced significant deformation by 67 GPa, and continued to deform to failure by 185 GPa. b) For C161 with a shape 2 profile and 15 µm torus depth, the anvils still experienced deformation on the shoulders and the culets cupped significantly, leading to failure by 186 GPa. c) C164 with a Shape 2 anvil and 18 µm torus depth showed significant improvement in the deformation of the anvils under pressure, but the anvils still failed below 2 Mbar seemingly due to cupping of the culets. Note that the anvil profiles provided do not scale with the absorption scans and are simply provided for reference to the anvil dimensions.


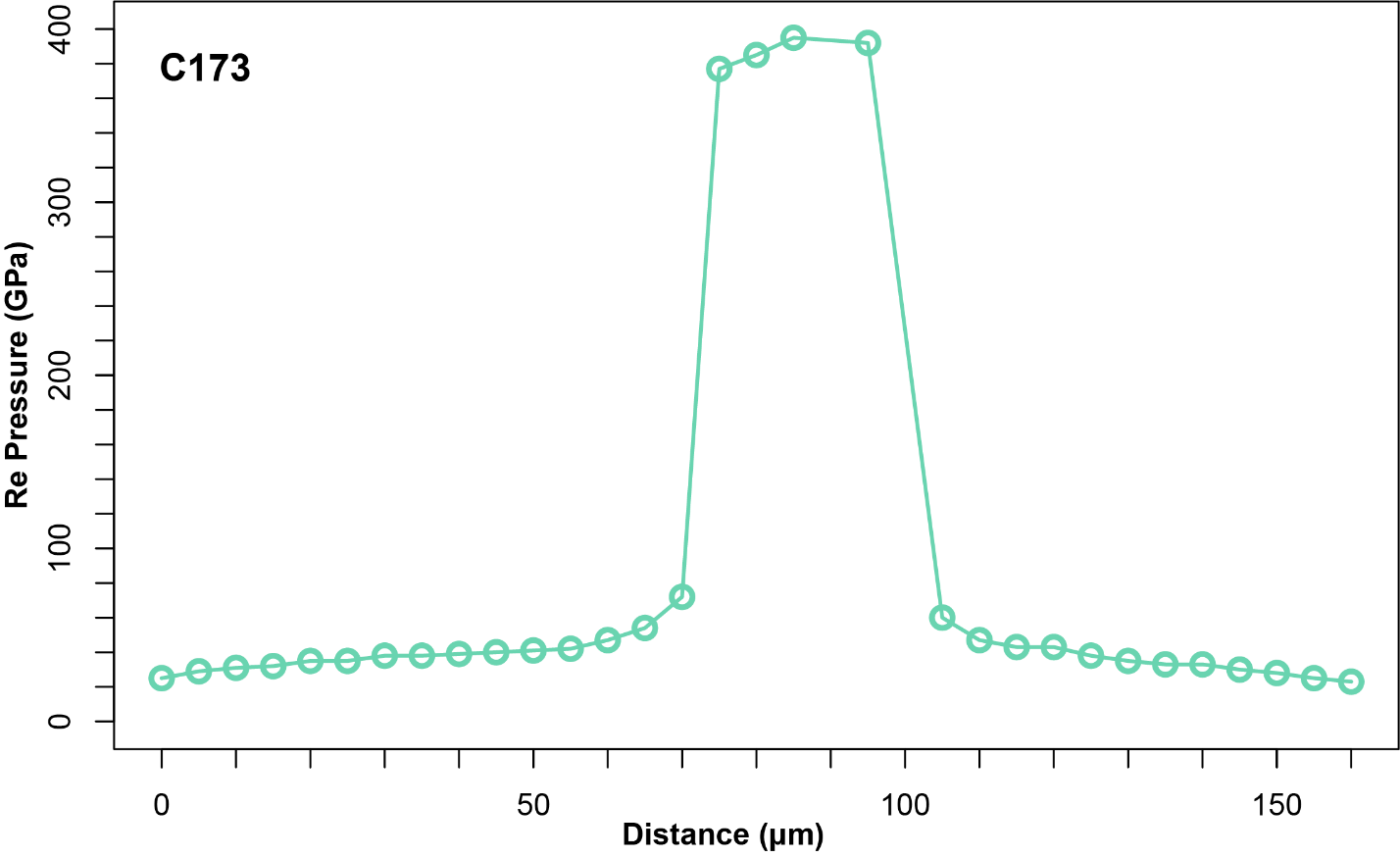


**Figure S4.** Pressure measured along the rhenium gasket across 160 µm of the sample when the center of the anvils measured a pressure from platinum of 367.4(4) GPa.^4^ The pressures shown in this cross-sectional scan were determined from Rhenium diffraction for uniform comparison of the pressure distribution across the toroidal anvil.^5^


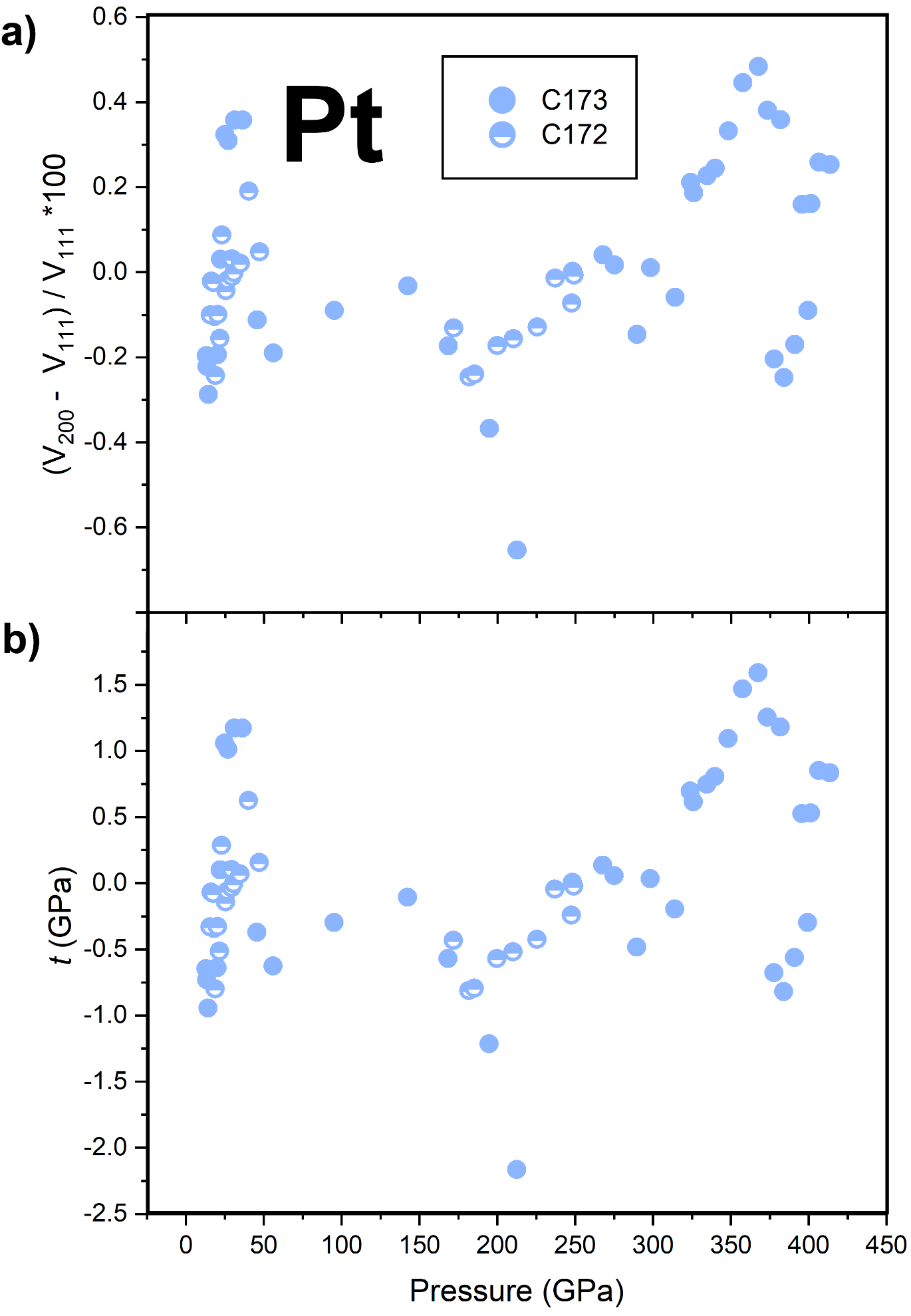


**Figure S5. a)** Percent volume residuals of the measured **for platinum** from the (200) lattice plane compared to the (111) plane across pressure range of this study. b) Uniaxial stress values calculated for Platinum based on the difference in volumes measured between the (111) and (200) lattice planes following the model discussed in Supplementary Text S4.


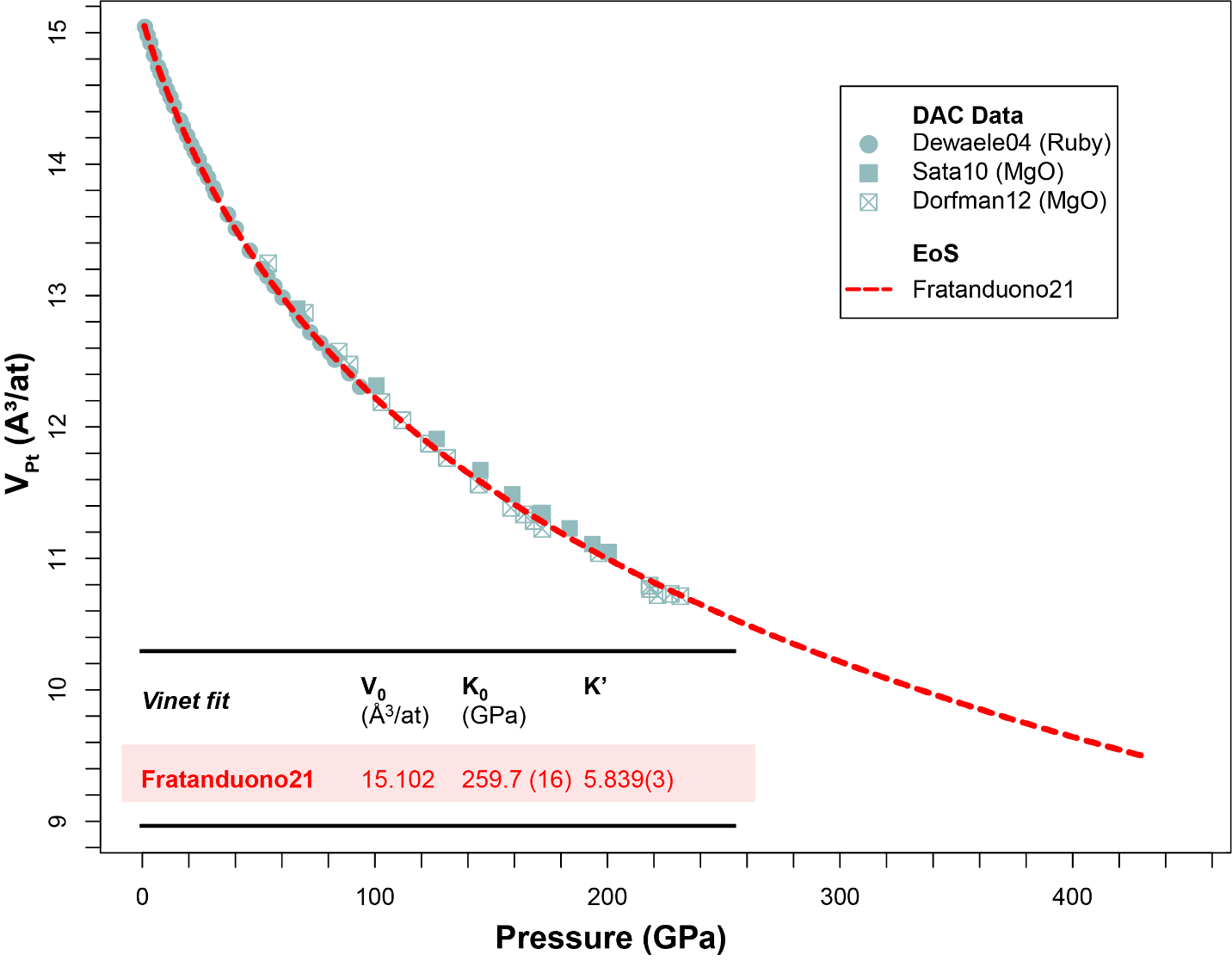


**Figure S6.** Comparisons of the DAC platinum data from (6) calibrated to ruby; (7) calibrated to MgO; and (8) calibrated to MgO and the ramp compression platinum scale.^4^

**Supplemental References**

1. Singh, A.K. The lattice strains in a specimen (cubic system) compressed nonhydrostatically in an opposed anvil device. *J. Appl. Phys.* **73**, 4278-4286 (1993).
2. Menéndez-Proupin, E. & Singh, A.K. Ab initio calculations of elastic properties of compressed Pt. *Phys.l Rev. B.*, **76**, 054117 (2007).
3. Takemura, K. & Dewaele, A. Isothermal equation of state for gold with a He-pressure medium. *Phys. Rev. B.* **78,** 104119 (2008).
4. Fratanduono, D.E., Millot, M., Braun, D.G., Ali, S.J., Fernandez-Pañella, A., Seagle, C.T., Davis, J.P., Brown, J.L., Akahama, Y., Kraus, R.G. & Marshall, M.C. Establishing gold and platinum standards to 1 terapascal using shockless compression. *Science* **372**, 1063-1068 (2021).
5. Anzellini, S., Dewaele, A., Occelli, F., Loubeyre, P. & Mezouar, M. Equation of state of rhenium and application for ultra high pressure calibration. *J. Appl. Phys.* **115**, 043511 (2014).
6. Dewaele, A., Loubeyre, P. & Mezouar, M. Equations of state of six metals above 94 GPa. *Phys. Rev. B* **70**, 094112 (2004).
7. Sata, N., Hirose, K., Shen, G., Nakajima, Y., Ohishi, Y. and Hirao, N. Compression of FeSi, Fe_3_C, Fe_0.95_O, & FeS under the core pressures and implication for light element in the Earth's core. *J. Geophys. Res: Solid Earth*, **115** (2010).
8. Dorfman, S.M., Prakapenka, V.B., Meng, Y. & Duffy, T.S. Intercomparison of pressure standards (Au, Pt, Mo, MgO, NaCl and Ne) to 2.5 Mbar. *J. Geophys. Res: Solid Earth* **117**, B08210 (2012).
